# Dissecting the phenotypic variability of osteogenesis imperfecta

**DOI:** 10.1242/dmm.049398

**Published:** 2022-05-16

**Authors:** Nadia Garibaldi, Roberta Besio, Raymond Dalgleish, Simona Villani, Aileen M. Barnes, Joan C. Marini, Antonella Forlino

**Affiliations:** 1 Department of Molecular Medicine, Biochemistry Unit, University of Pavia, 27100 Pavia, Italy; 2 Department of Genetics and Genome Biology, University of Leicester, Leicester LE1 7RH, United Kingdom; 3 Department of Public Health and Experimental and Forensic Medicine, Unit of Biostatistics and Clinical Epidemiology, University of Pavia, 27100 Pavia, Italy; 4 Section on Heritable Disorders of Bone and Extracellular Matrix, NICHD, NIH, 20892 Bethesda, MD, USA

**Keywords:** Bone, Osteogenesis imperfecta, Phenotypic variability

## Abstract

Osteogenesis imperfecta (OI) is a heterogeneous family of collagen type I-related diseases characterized by bone fragility. OI is most commonly caused by single-nucleotide substitutions that replace glycine residues or exon splicing defects in the *COL1A1* and *COL1A2* genes that encode the α1(I) and α2(I) collagen chains. Mutant collagen is partially retained intracellularly, impairing cell homeostasis. Upon secretion, it assembles in disorganized fibrils, altering mineralization. OI is characterized by a wide range of clinical outcomes, even in the presence of identical sequence variants. Given the heterotrimeric nature of collagen I, its amino acid composition and the peculiarity of its folding, several causes may underlie the phenotypic variability of OI. A deep analysis of entries regarding glycine and splice site collagen substitution of the largest publicly available patient database reveals a higher risk of lethal phenotype for carriers of variants in α1(I) than in α2(I) chain. However, splice site variants are predominantly associated with lethal phenotype when they occur in *COL1A2*. In addition, lethality is increased when mutations occur in regions of importance for extracellular matrix interactions. Both extracellular and intracellular determinants of OI clinical severity are discussed in light of the findings from *in vitro* and *in vivo* OI models. Combined with meticulous tracking of clinical cases via a publicly available database, the available OI animal models have proven to be a unique tool to shed light on new modulators of phenotype determination for this rare heterogeneous disease.

## Abstract (precis)

**Summary:** This Clinical Puzzle analyses the largest public OI database as well as data from animal models to address how type I collagen defects contribute to the phenotypic variability of the brittle bone disease osteogenesis imperfecta.

## Introduction

Osteogenesis imperfecta (OI), often referred to as brittle bone disease, is a family of skeletal disorders characterized by bone deformity, bone fragility and limb and vertebral fractures (Marini et al., 2017). Its incidence of 1 in 10–20,000 live births classifies OI as a rare disease, although it represents one of the most frequent of the rare skeletal disorders (Marini and Dang Do, 2000). Extra-skeletal phenotypes, including disordered tooth development (dentinogenesis imperfecta), blue sclerae, skin and joint laxity, muscle weakness and cardiopulmonary impairment, also occur in patients with variable frequency. Thus, OI is a generalized connective tissue disorder rather than a purely skeletal one.

To date, 22 different OI types are included in the OMIM database, referred to as OI type I to XXII (Table 1), with the OI scientific community still debating this disease classification (Forlino and Marini, 2016; Sillence and Rimoin, 1978; Sillence et al., 1979; Van Dijk and Sillence, 2014; Tournis and Dede, 2018; Mortier at al., 2019; Chetty et al., 2020). The majority of individuals with OI (85–90%) harbor dominant variants in the *COL1A1* (HGNC:2197) and *COL1A2* (HGNC:2198) genes, encoding the α1 and α2 chains of collagen type I, and are classified as having classic OI types I to IV (Marini et al., 2017). Since 2006, the molecular characterization of murine models with fragile bone phenotypes, as well as the technical advance, cost reduction and accessibility of next-generation sequencing, have allowed the identification of several other causative OI genes in individuals with clinical features but without collagen I molecular defects. These newly described variants have mainly recessive inheritance, with the exception of those in interferon-induced transmembrane protein 5 (*IFITM5,* HGNC:16644) and membrane-bound transcription factor peptidase, site 2 (*MBTPS2,* HGNC:15455) genes, which have autosomal dominant and X-linked recessive transmission, respectively (Besio et al., 2019a). The variants in the non-collagen causative genes are responsible for impaired collagen synthesis, post-translational modification, secretion and processing, or for compromised osteoblast function (Etich et al., 2020; Marini et al., 2017).

**Table 1.**
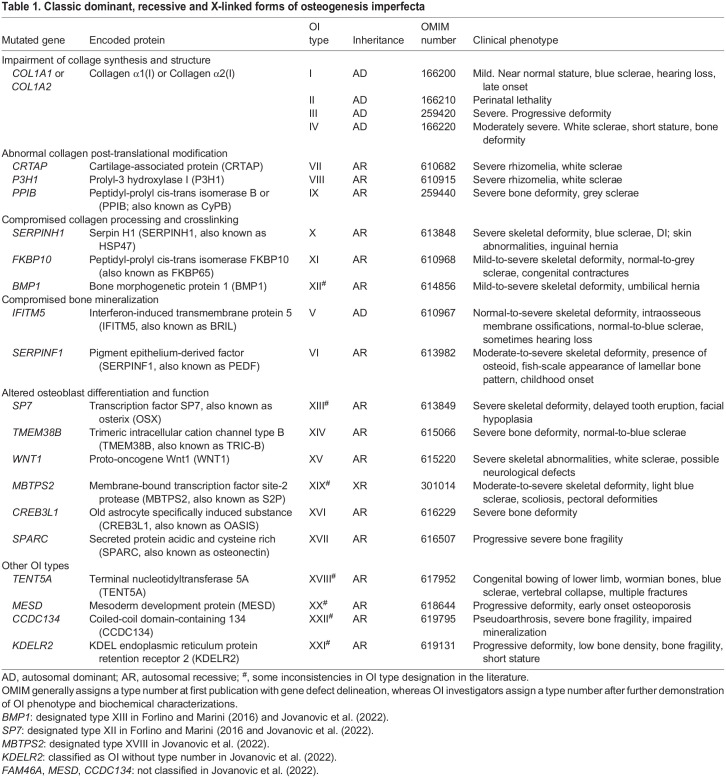
Classic dominant, recessive and X-linked forms of osteogenesis imperfecta

Thus, regardless of the mutated protein, and considering the biochemical effect of the defective molecules, OI is a collagen type I-related disorder. Type I collagen is the most abundant structural protein in humans and synthesized in the endoplasmic reticulum (ER) as a heterotrimeric precursor of two α1 chains and one α2 chain. Each procollagen α chain contains a central triple-helix region of 1014 amino acids, characterized by 338 uninterrupted Gly-Xaa-Yaa repeats, linked to globular N- and C- terminal propeptides through short non helical telopeptides (Gelse et al., 2003; Holmes et al., 2017; Marini et al., 2017). The fact that glycine represents every third amino acid in the triple helix is crucial for correct chain folding (Prockop and Kivirikko, 1995).

Two procollagen chains, i.e. proα1(I) and one proα2(I), are synthesized in the lumen of the ER and form a [proα1(I)_2_proα2(I)] molecule (Canty and Kadler, 2005). During the folding process, specific post-translational modifications occur (Canty and Kadler, 2005; Krane, 2008). Several chaperones collaborate in chain folding and favor trimer stabilization, supporting procollagen secretion through COPII vesicles. In the extracellular space, the peptidases ADAM metallopeptidase with thrombospondin type 1 motif 2 (ADAMTS2) and bone morphogenic protein 1 (BMP1) cleave the N- and C-terminal propeptides, respectively, promoting the spontaneous self-assembly of collagen fibrils that are then stabilized by enzyme-driven covalent crosslinks (Canty and Kadler, 2005).

Classic OI can arise due to quantitative as well as qualitative defects in collagen type I. Sequence variants in *COL1A1* causing haploinsufficiency halve the amount of an otherwise normal protein, resulting in extracellular matrix (ECM) deficiency. This generally results in the mildest OI type I phenotype, characterized by mild osteoporotic bone, near-normal stature and secondary features, such as blue sclerae and late-onset hearing loss (Willing et al., 1996).

**Fig. 1.**
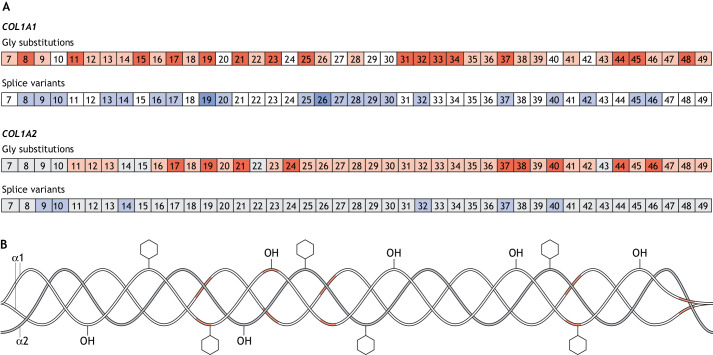
**Lethal regions in collagen type I.** (A) Exons containing glycine substitutions (red) and splice variants (blue) in *COL1A1* (top) and *COL1A2* (bottom) that are associated with phenotypic variability*.* Exons of which variants have been identified in >20 affected individuals are indicated by a darker shade of red and blue. Exons without reported sequence variants are in white for *COL1A1* and in grey for *COL1A2*. (B) Structure of collagen type I protein showing the regions associated with lethal mutations in α1(I) in red.

A much wider phenotypic variability is associated with *COL1A1* and *COL1A2* sequence variants that are responsible for type I collagen structural defects, ranging from the aforementioned mild (type I), moderate (type IV), progressively deforming (type III) to lethal (type II) OI. Single-nucleotide substitutions in which glycine residues are replaced with bulkier amino acids (hereafter referred to as glycine substitutions) are the most frequent, followed by changes in 5′ or 3′ splice sites – resulting in exon skipping – or the use of cryptic splice sites (Fig. 1) (Marini et al., 2007). Less frequently, duplications or deletions, sometimes affecting one or two Gly-Xaa-Yaa triplets, which shift inter-chain registers, and sequence variants in the procollagen C-propeptide, which hamper chain association, folding and processing, have also been reported (Barnes et al., 2019; Cabral et al., 2003; Symoens et al., 2014). These variants delay α chain recognition and/or helical folding, thereby allowing excessive post-translational modifications, which results in an altered molecular structure that exerts a dominant-negative effect on collagen fibril formation and function (Forlino and Marini, 2016).

Phenotypic variability in classic OI can be due to the affected collagen chain gene, the type and the position of the variant within the affected gene, the chemical nature of the replacing amino acid or, more likely, a combination of the above (Forlino et al., 2011). Interestingly, identical sequence variants can cause different phenotypes, making counselling for OI extremely complex. For splice site mutations in particular, it should be considered that splicing is an immensely complex process involving several RNAs and proteins. There is evidence that *COL1A1* and *COL1A2* splicing outcomes may be affected by variants in genes that encode splicing components (Mucaki et al., 2020), suggesting the existence of modifier genes in OI. In addition, sequence variants close to, but not immediately next to the mutated splice site, may also contribute to phenotypic variation between individuals harboring the same splice-site sequence variant. Furthermore, the presence and the effects of gene modifiers cannot be excluded in OI caused by glycine substitution.

Understanding the genotype-phenotype relationship in OI is still a debated issue in the field (Marini and Dang Do, 2000; Marini et al., 2007; Zhytnik et al., 2020). In this Clinical Puzzle, we analyze the largest publicly available database for OI, which includes genetic and phenotypic descriptions for most entries; we also discuss the possible molecular basis underlying phenotypic variability in the classic forms of the disease (https://lovd.nl/OI-genes) (Dalgleish, 1997, 1998). We focus on the most-common sequence variants found in the collagen triple-helix region, a structure that is crucial for fibrillogenesis and for the correct interaction with cells and other ECM proteins. We also present a critical overview of emerging *in vitro* data, as well as of the dominant OI murine and zebrafish models, to facilitate the understanding of the link between the molecular defect and the clinical OI phenotype.

## Patient datasets and statistical analyses

For our retrospective cohort analysis, we used the osteogenesis imperfecta variant database (https://databases.lovd.nl/shared/genes/COL1A1; https://databases.lovd.nl/shared/genes/COL1A2). At the time of analysis (July 2021), this database included over 3000 (*n*=3152) individuals diagnosed with OI, of whom 2169 carry glycine substitutions, splice site sequence variants, deletions and insertions in *COL1A1* and 983 in *COL1A2*. Single-nucleotide variants within the triple-helical region of α1(I) (*n*=1009) and α2(I) (*n*=762) chains that cause glycine substitutions and splice site defects in coding exons (exons 7 to 48 for *COLA1* and to 49 for *COL1A2*) were considered in the analysis and correlated with patient phenotypes. All variants are class 5 pathogenic according to the American College of Medical Genetics and Genomics guidelines. The splice site analysis only included variants within the most representative sites, i.e. the highly conserved donor splice sites (+1, +2 positions) and the acceptor splice sites (−1, −2 positions). Our phenotypic variability analysis excluded those clinical cases for which no OI type was reported, individuals with sequence variants in the N- and C- telo- or pro-peptides, those with insertions, duplications or deletions, and those with intronic defects other than the acceptor and donor splice sites at the first two or last two positions.

When describing disease severity, we followed the Sillence classification of OI type I- IV reported in the database for each individual, without additional clinical verification (Van Dijk and Sillence, 2014). Taking these subjective features into account as well as the available information regarding each individual at the time of diagnosis, statistical analysis was performed after grouping individuals with lethal OI (type II) and non-lethal OI (types I, III, and IV). To indicate individual variants, we used both the legacy amino acid numbering system for collagen chains, which designates the first glycine of the triple-helical region as amino acid 1, and the systematic numbering of the glycine position starting at the AUG start codon. For the α1 chain, there are178 fewer legacy than systematic amino acid numbers in the triple-helical region. For the α2 chain, there are 90 fewer legacy amino acid numbers.

In our retrospective cohort study, individuals with α1(I) chain variants were the exposed cohort and those with α2(I) chain variants the reference category. The lethal phenotype occurrence was the outcome of interest. Cumulative incidence of genotypes causing lethal outcomes was estimated by α chain variants. The association between position and type of the substituting amino acid was investigated using chi-squared or Fisher's exact tests. Unconditional logistic regression was applied to evaluate the effect of the α chain variant on lethal phenotype occurrence (dependent variable) adjusting for position and type of the replacing amino acid as prognostic factors or potential confounder. The adjusted effect of chain variants on lethal phenotype was expressed as odds ratio (OR) with 95% confidence interval (95% CI). *P*<0.05 was considered significant (two-sided). All analyses were carried out by using STATA 15 (StataCorp, 2017).

## OI phenotypic variability: effects of the gene, type of mutation and nature of the substituting amino acid

Given the heterotrimeric nature of type I collagen, its amino acid composition and the peculiarity of its folding process, the phenotypic variability in OI might have several explanations. The specific chain is the first variable that needs to be considered, followed by the position and type of the mutation along that chain, and the nature of the amino acid replacing glycine (Box 1).

### Mutated gene: *COL1A1* versus *COL1A2*

**Fig. 2.**
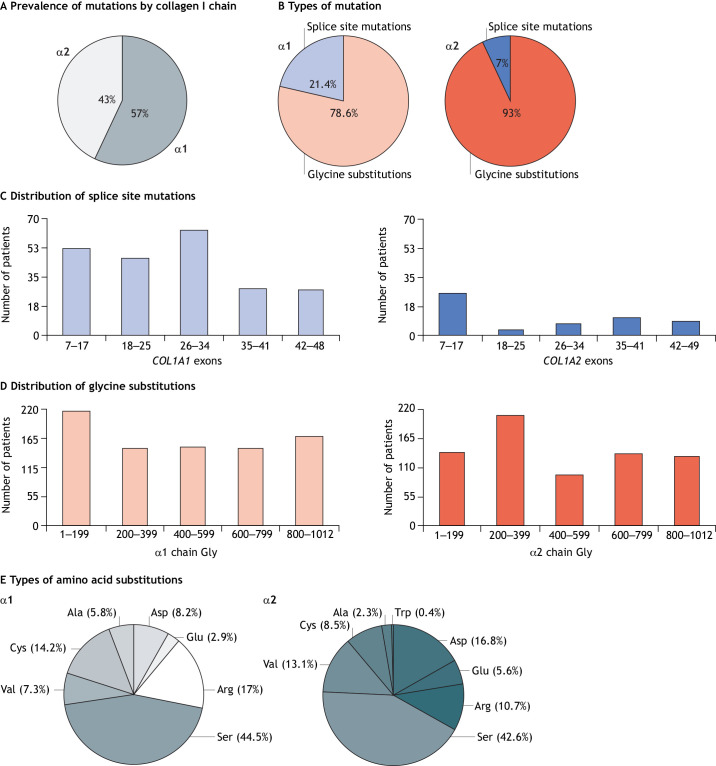
**Distribution of collagen type I sequence variants in α1(I) and α2(I) chains, type/position of the variants and nature of the substituting amino acid in osteogenesis imperfecta (OI).** (A) Overall distribution of sequence variants in collagen I chains. (B) Distribution of glycine substitutions and splice site sequence variations in the α1(I) and α2(I) chains of collagen I. Glycine substitutions are shown in red, splice site mutations are shown in blue. (C) Distribution of splice site sequence variants along the *COL1A1* and *COL1A2* genes. (D) Distribution of glycine (Gly) substitution sequence variants along the α1(I) and α2(I) chains. (E) Distributions of bulkier amino acids substituting glycine in the α1(I) and α2(I) chains, resulting in structure alterations. These panels summarize data from our new analysis of the public *COL1A1* and *COL1A2* sequence variant databases. We focus on OI causative variants that affect the triple-helix region of collagen type I. *COL1A1*: https://databases.lovd.nl/shared/genes/COL1A1; *COL1A2*: https://databases.lovd.nl/shared/genes/COL1A2.

Our analysis collated 2105 OI type-independent single-nucleotide substitutions or exon splicing events in the helical domains of proα1(I) and proα2(I), more than doubling the dataset of the last comprehensive genotype/phenotype analysis previously performed (Marini et al., 2007). Among these single-nucleotide changes, 1271 (60.4%) were in *COL1A1* and 834 (39.6%) in *COL1A2,* providing enough information to update the previous hypotheses regarding the molecular basis of OI phenotypic variability. This present analysis is limited to the 1771 (84.1%) individuals with OI for whom a clear Sillence clinical classification was reported in the database; 1009 (57%) cases with sequence variants in *COL1A1* and 762 (43%) in *COL1A2* (Fig. 2A).

**Box 1. Clinical case presentation: different phenotypes caused by identical or different mutations**

*COL1A1* NM_000088.3:c.2299G>A causing Gly589Ser, p.Gly767Ser – three individuals all carrying a serine for glycine substitution show different phenotypes

**Patient 1** (panels A-D) showed severe OI type III. He was born at 34 weeks gestation by breech spontaneous vaginal delivery. As a newborn, this patient was found to have multiple healed *in utero* fractures and new fractures, and light blue sclerae. His limbs were short and severely bowed, with radiographic ‘popcorn’ formation at the distal femoral joint**.** He incurred dozens of long bone fractures and Bailey rods were placed in his femurs. Although he was able to walk short distances when aged between 4 and 9 years, he subsequently used a wheelchair for mobility. By age 9 years, he had S-curve scoliosis and a barrel chest; all vertebrae were biconcave. The patient had dentinogenesis imperfecta with yellow-brown discoloration of his teeth and class III malocclusion. His pelvis had severe *protrusio acetabuli* and deformity of the iliac wings. At age 13 years, his height was in the 50th percentile for a 4-year-old boy. The patient had macrocephaly (+2 standard deviations for an average adult male) and CT scans showed odontoid compression of the medulla. He died at age 29 from pneumonia (Marini et al., 2007).

**Patient 2** (panels E-G) has moderate to severe OI type IV. His OI diagnosis was made on day 13 of life with notation of birth fractures of femurs and ribs. He had dentinogenesis imperfecta. His recurrent femoral fractures were managed with fixation by plates and screws at age 5 years, replaced with intramedullary rods at age 10 years. He attained ambulation with bracing, then transitioned to crutches. The patient retained ambulation in his household and used a motorized wheelchair in the community. His height at age 5 years was 50th centile for 3-year-old boys, and at age 18 years was 50th centile for a 9-year-old. Scoliosis first developed around age 13 years, along with multiple vertebral compression deformities, and progressed through his teen years. His DXA L1-L4 z-score was −4.89 at 12 years of age, indicating a severe low bone mass. A normal echocardiogram, normal audiology and mild restrictive lung disease were noticed at age 15 years (Marini et al., 2007).

**Patient 3 (**panels H and I) has mild OI type IV. She was delivered by C-section due to breech. Bone deformity was noticed on radiographs of the newborn that also demonstrated a new humeral fracture. When examined at 5 months of age, she had a healing tibial fracture. The patient was small, with length at the 50th centile for a 2-month-old girl. Her motor development was normal. She continued to develop well and was an independent ambulator (J.C.M. and A.M.B., unpublished).

*COL1A1* NM_000088.3:c.976G>C causing Gly148Asp, p.Gly326Asp and *COL1A1* NM_000088.3:c.977G>A causing Gly148Arg, p.Gly326Arg – substitution of the same glycine residue for different amino acids causes different phenotypes

**Patient 4** (panels J-L) is a 20-year-old male with *COL1A1* Gly148Asp substitution causing OI type IV. He was small for his gestational age and had been delivered by C-section for breech. He had bilateral inguinal hernias and bilateral fractures of tibia and femora. At initial examination at age 2 years, height was that of a 3-month-old and weight that of an average 5-month-old. The patient had dentinogenesis imperfecta, with translucent grey teeth. His fracture history includes multiple femoral and several tibial fractures. He underwent multiple lower extremity osteotomies and rod placements/replacements in all four long bones. He attained ambulation when ∼3 years of age, using shoe orthotics to correct leg-length discrepancy. At age 3.5 years, his DXA L1-L4 z-score was −5.1, indicating significant vertebral bone density loss. The patient experienced multiple compression fractures in vertebrae, and mild scoliosis first appeared at ∼7 years of age. He received cycles of pamidronate every 3 months when aged between 5 and 9 years, resulting in lumbar spine DXA z-score improvement by 1.3 standard deviations and improved vertebral geometry. At age 20 years, his height was the 50th centile for a 7.5-year-old boy; his DXA L1-L4 z-score was −6.6, echocardiogram revealed dilated aortic root, spirometry revealed a pattern of moderate restrictive lung function and hearing loss was stable. The patient used a cane as a mobility aide (Marini et al., 2007). The same *COL1A1* Gly148Arg substitution had also been identified in a third-generation pedigree with multiple affected members (J.C.M., unpublished). All affected individuals had one or more long bone fractures in childhood and adulthood. Owing to their relatively moderate phenotype, these individuals might not have come to diagnosis individually, were they not members of a pedigree with common findings. Their heights are at the shorter end of the normal range and there is no long bone deformity or scoliosis. Their DXA z-scores are in the osteopenic range and sclerae are light blue. Taken together, the phenotype in this family is similar to that of type I OI.

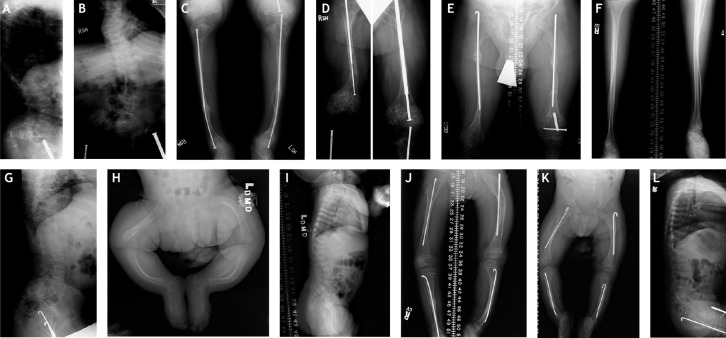

**X-rays of patients 1, 2, 3 and 4 with OI show phenotypic variability associated with the same glycine substitution with identical or different bulkier amino acids.** (A-D) Radiographs of patient 1, taken at 13 years 2 months. (E-G) Radiographs of patient 2, taken at 15.5 years. (H,I) Radiographs of patient 3, taken at 5.5 months. (J-L) Radiographs of patient 4, taken at 5 years 7 months (J), 2 years 10 months (K) and at 4 years (L).

All radiographs were taken at the NIH Clinical Center following internal review board-approved protocols and none have previously been published.

**Fig. 3.**
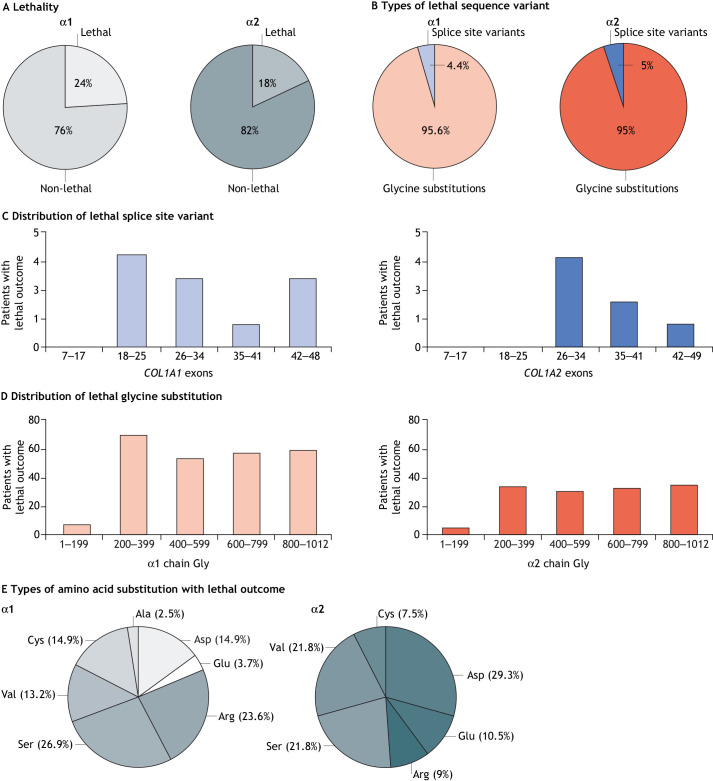
**Effects of collagen type I sequence variants in α1(I) and α2(I) chains on OI lethality.** (A) Overall distribution of sequence variants in collagen I chains that cause lethal OI. (B) Distribution of lethality between glycine substitution and splice site sequence variants in α1(I) and α2(I) chains. (C) Frequencies of lethal outcomes associated with splice site sequence variants along the *COL1A1* and *COL1A2* genes. (D) Frequencies of lethal outcomes associated with glycine substitutions along the α1(I) and α2(I) chains. (E) Distributions of glycine-substituting amino acids in α1(I) and α2(I) chains that are associated with a lethal outcome. These panels summarize data from our new analysis of the public *COL1A1* and *COL1A2* sequence variant databases. We focus on OI causative variants that affect the triple-helix region of collagen I. *COL1A1*: https://databases.lovd.nl/shared/genes/COL1A1; *COL1A2*: https://databases.lovd.nl/shared/genes/COL1A2.

Lethality was reported for 392 (22.1% of the total) individuals with OI, of which 252 carried variants in *COL1A1,* representing 24.9% of all reported variants in that gene and 140 carried variants in *COL1A2* (18.4%) (Fig. 3A). Adjusting for the type of amino acid substitutes and their positions, the risk of lethal phenotype is greater for carriers of α1(I) chain variants than for α2(I) chain variants (OR=4.7, 95% CI [3.37-6.55] *P*<0.001), probably because of collagen type I stoichiometry. In other words, the effect of α chain variants on lethal genotype initially detected in the 2007 report (Marini et al., 2007) is, indeed, significant, and independent of the substituting amino acid and its position.

### Type of variant: glycine substitution versus exon splicing

Among the variants in the *COL1A1* sequence that encode the collagen I triple helix (Fig. 1), we identified 793 (78.6%) glycine substitutions and 216 (21.4%) splice site changes at position ±1 or ±2 responsible for altered exon splicing, whereas in *COL1A2,* we annotated 709 (93.0%) glycine substitutions and 53 (7.0%) splice site changes (Fig. 2B).

We found even distribution of first-position glycine substitutions in the α1(I) (217/338, 64.2%) and α2(I) (222/338, 65.7%) collagen tripeptide units (Gly-Xaa-Yaa). In contrast, of the 84 possible splice sites in the triple-helix region of *COL1A1,* 67 (79.7%) were mutated, whereas of the 86 possible sites in *COL1A2*, only 22 (25.5%) were affected, making intronic defects more frequent in *COL1A1* compared with *COL1A2.*

Regarding lethal sequence variants, of the 252 reported in α1(I), 241 (95.6%) were glycine substitutions, whereas 11 (4.4%) were splice site variants; in α2(I), 133 of 140 (95%) were glycine substitutions and 7 (5%) splice site variants (Fig. 3B). Of individuals with lethal OI due to splice site mutations, more than 50% carry variants in *COL1A2* (7 of 53, 13.2%) as compared with *COL1A1* variants (11 of 216, 5%). Indeed, the occurrence of a lethal phenotype is significantly reduced for splice defects in the α1(I) chain compared with that in the α2(I) chain (5.1% vs 13.2%, Fisher's exact test *P*=0.042).

Taken together, our analyses shows that splice site variants are less frequent than glycine substitutions and more prevalent in *COL1A1*. Nevertheless, exon splicing defects yield a higher number of severe phenotypes when they occur in *COL1A2*.

### Position of mutations in genes and chains, and their effects

To understand the phenotypic effect of the sequence variants according to their position along the chains, *COL1A1* and *COL1A2* were divided in five equally sized regions based on exons (i.e. 7–17, 18–25, 26–34, 35–41 and 42–49) corresponding to five regions along the α1 and α2 chains comprising legacy amino acid numbers (i.e. 1–199, 200–399, 400–599, 600–799 and 800–1012), because amino acid numbering in the performed analysis referring to glycine 1 as the first glycine of the triple-helix region (Fig. 1).

The 216 identified splice site variants of *COL1A1* are distributed along the gene, with the majority falling in the first three quintiles. In contrast, of the 53 splice defects of *COL1A2*, more than half are in the first quintile (Fig. 2C). Nevertheless, none of the lethal variants occur in the first 200 amino acids (Fig. 3C). Lethality linked to *COL1A1* occurs due to splice site variants within exon 20 up to the 3′ end of the transcript, whereas in *COL1A2* lethality occurs from exon 28 onwards.

The identified glycine substitutions are evenly distributed along both α(I) chains (Fig. 2D). Of the 793 patients with α1(I) glycine substitutions, 241 (30.4%) carried lethal variants. These were also equally distributed along the chain, except for those of the first quintile, where only few glycine substitutions were linked to individuals with the lethal OI type II (Fig. 3D). In a previous analysis, we reported very few individuals, who had been diagnosed with non-lethal OI and were carrying variants in α1(I) major ligand-binding regions 2 and 3 (MLBR2 and MLBR3, respectively), both of which being important for interactions with non-collagenous ECM proteins (Marini et al., 2007). In our expanded dataset, of the 53 individuals with OI reported to carry variants in MLBR2 (aa 682–830), 30 (56.6%) had a lethal phenotype; of the 55 individuals with OI carrying variants in MLBR3 (aa 920–1012), 25 (45.4%) also had a lethal phenotype.

Only 133 of the 709 individuals (18.7%) with glycine substitutions in the α2(I) chain had a lethal phenotype, with very few cases linked to substitutions of the first 200 amino acids (Fig. 3D) and the remainder comprising approximately one-third of the total lethal cases. Our aforementioned 2007 study reported the localization of lethal sequence variants in eight evenly spaced regions along the α2(I) chain, crossing the exon boundaries defined above (Marini et al., 2007). These regions are important for extracellular interactions of collagen with proteoglycans. By using the regional boundaries previously proposed, our analysis included sufficient individuals with non-lethal OI, allowing us to discern that average lethality due to mutations within these individual regions is roughly the same as the average lethality of variants across the entire α2(I) chain. However, we also identified shorter stretches of glycine substitutions in α2(I) within seven of the previously proposed regions linked to the 56–66% of individuals with lethal OI type II and/or the severest non-lethal phenotypes described as OI type II/III. This enrichment of lethal phenotypes within localized areas at roughly twice the average as compared with that of the entire α chain indicates that the crucial interactions of collagen with non-collagenous molecules involve discrete residues within their binding sites, for which further investigation is needed. This level of lethality, with preponderance rather than exclusivity, within α chain regions prevents them from being used confidently when counselling families regarding a phenotypic outcome.

### OI phenotypic variability: effect of the glycine-substituting amino acid

The polar amino acid serine (Ser) is the most frequent substitute in both α(I) chains, accounting for 44.5% of glycine substitutions in α1(I) and 42.6% in α2(I) (Fig. 2E).

Interestingly, analyzing the frequency of lethality associated with different substituting amino acids revealed that, in α1(I), Ser is the substitution most frequently (26.9%) associated with OI type II, followed by Asp and Cys (14.9% each), Val (13.2%), Glu (3.7%) and Ala (2.5%). In α2(I), the substitution accounting for the most individuals with lethal OI is Asp (29.3%), followed by Ser (21.8%) and Val (21.8%), Glu (10.5%), Arg (9%) and Cys (7.5%) (Fig. 3E). The polar Ser and the charged Asp account for ∼50% of lethality frequency in both chains.

It is known that mutation of the same glycine residue frequently results in phenotypic variability. In the α1(I) chain, 97 (28.7%) of the 338 glycine residues that are replaced by bulkier amino acids have been associated with phenotypic variability in at least two patients. Among these substitutions, 39 (40.2%) were responsible for variable non-lethal outcomes (OI types I, III and IV) and 58 (59.8%) include at least one lethal case. In the α2(I) chain, 112 (33.1%) of the 338 glycine residues substituted for bulkier amino acids in at least two individuals have been associated with phenotypic variability and, among them, 63 (56.2%) were responsible for non-lethal outcomes, ranging from OI I to III  and to IV, whereas 49 (43.7%) also included a lethal outcome.

We identified 79 (23.4%) glycine positions in the α1(I) chain for which all carriers presented with a lethal phenotype; however, this lethal outcome was only observed in less than half, i.e. 37 (10.9%), individuals when glycine substitutions at these positions occurred in the α2(I) chain. These substitutions were distributed along both chains, except for the first N-terminal 200 amino acids of the triple-helix region of either chain, in which no such lethal outcome in individuals with OI was identified.

A challenge in understanding OI variability are the different phenotypes that result from replacement of the same glycine residue with a different or even the same amino acid. To address this challenge, the various mutational hotspots – namely glycine substitutions that have ≥10 individuals with OI listed in the database – represent a precious tool. Also, concordance between genotype and phenotype is greater for substitutions in α2(I) than in α1(I). As mentioned above, hotspots upstream of the N-terminal 200 amino acids of the triple-helix region present only a non-lethal phenotype. Substitutions at amino acid positions 200–399 and after position 800 are linked to lethal phenotype at significantly higher frequencies in α1(I) relative to the α2(I) chain (41.82% vs 5.77%, chi-squared test =31.35 *P*<0.001 relative to the 18.39% vs 0%, Fisher's exact test *P*=0.003, respectively).

### OI phenotypic variability: effect of exon splicing

In *COL1A1,* 25 of the 84 splice site positions (29.7%) were associated with phenotype variability. Of these, 21 (84%) were reported in non-lethal OI of varying severity, while four (16%) included at least one patient with lethal OI. Only four (4.7%) were exclusively linked to a lethal phenotype and only three splice site mutation hotspots were identified in more than ten individuals – all in 5′ splice sites and all associated with non-lethal outcomes.

In *COL1A2*, eight of the possible 86 splice site positions (9.3%) were associated with phenotypic variability. Six in particular, were linked to differing non-lethal outcomes; a further two included individuals with lethal OI. Only five (5.7%) positions presented uniquely with a lethal phenotype.

In collagen genes, exons encode a discrete number of Gly-Xaa-Yaa tripeptides, meaning that, with respect to each other, exon splicing results in a register shift of the collagen chains within the helix. Thus, splicing variants cause abnormal collagen assembly, stability and incorporation into fibrils, overall impairing matrix mineralization. Moreover, the clinical outcome, probably depending on the resulting register-shifted triple helix, can be affected.

### Gap regions and phenotypic variability

We previously have identified regions in both α1(I) and α2(I) chains that lack any reported variants of glycine substitution, hypothesizing that these gaps are associated with either very mild, thus undetectable, phenotypes or very severe ones that cause premature embryonic death (Marini et al., 2007). The increased number of individuals with OI recorded in the database has shed further light on these regions. Lethal variants have, indeed, been confirmed in all four previously identified gaps of seven or more glycine residues within the α1(I) chain, namely 328–346, 418–436, 484–505 and 805–820, supporting a relevant role of these sites for collagen structure and interaction with extracellular proteins. In contrast, only one of the five gaps previously recognized in α2(I) (Marini et al., 2007) is still characterized by an absence of reported sequence variants. This region, spanning amino acids 133–154, overlaps with the integrin-binding site in MLBR1. We also highlighted two long glycine stretches in the α1(I) chain with almost exclusively lethal outcome-linked sequence variants. These correlate with MLBR2 and MLBR3, spanning amino acids 691–823 and 910–964, respectively (Di Lullo et al., 2002; Marini et al., 2007). Our analysis confirmed that this MLBR3 region, indeed, corresponds to exclusively lethal phenotypes. We also confirm that amino acids 691–823 in MLBR2 still correlate to mainly lethal phenotypes but the exclusively lethal stretch in our analysis is shortened to nine triplets (799–823). Interestingly, the gap region in α1(I) that does not comprise any identified sequence variants does overlap with a specific binding site for α2β1 integrin and HSP47 (San Antonio et al., 2020). The regions previously proposed to be in the lethal region model for α2(I) are now associated with a sufficient number of individuals showing non-lethal OI in order to lower the lethality of this region to the α2(I) chain average, thus, rendering genetic counselling untenable. However, in seven out of eight previously described α2(I) regions are stretches of amino acid spanning 9–20 triplets that are associated with twice as many individuals showing the very severe OI types II and II/III, rather than the chain average. This implies that discrete residues in collagen I is crucial for proteoglycan binding and that variants of these residues cause particularly severe protein disfunction.

Overall, our analysis revealed more frequent and more lethal variants in *COL1A1* than in *COL1A2*, with a prevalence of glycine substitutions over exon splicing mutations. Lethal splicing mutations primarily affect the *COL1A1* gene. Importantly, analyses have confirmed that mutation among the first 200 amino acids of the triple-helix region are not associated with lethality, whereas mutations in MLBR2 and MLBR3 negatively modulate the clinical phenotype.

In both α(I) chains, Ser and Asp account for the most-lethal glycine-substituting residues.

Given the complexity of type and position of OI-causing mutations within collagen genes in the human genetic background, and of retrieving bone samples from individuals with OI for deep analysis, generation and characterization of animal models represents a research avenue that can be used to dissect phenotypic variability of this rare disease.

## Animal models of classic OI

A number of murine and zebrafish models of classic OI are available. These models carry glycine substitutions or splice site variants with dominant transmission in the endogenous collagen genes, providing useful tools to further dissect OI phenotypic variability (Chen et al., 2014; Daley et al., 2010; Fisher et al., 2003; Forlino et al., 1999; Gistelinck et al., 2018). These models guarantee a large number of organisms with defined, either outbred or inbred, genetic backgrounds. Moreover, they offer the possibility to evaluate the geometric and biomechanical properties of bones with sufficient statistical power, which is impossible when using patient bone. However, the small number of sequence variants reproduced in the available models is the main drawback that narrows their power.

The heterozygous *Col1a1*^+/G349C^ mouse, called *Brtl*, was the first non-lethal murine OI knock-in model. It carries an α1(I)-Gly349Cys mutation that had originally been identified in two probands with moderately severe OI and, subsequently, in one lethal case (Forlino et al., 1999; Sarafova et al., 1998; https://databases.lovd.nl/shared/genes/COL1A1). *Brtl* combines a typical OI glycine defect with the autosomal dominant inheritance of classic OI and the phenotypic variability described in some patients carrying identical sequence variants, but with different disease severity (Marini et al., 1993). Indeed, about 30–40% of *Brtl* mice die at birth because of respiratory stress. Radiographs and skeletal staining of lethal *Brtl* mice reveal a flared ribcage with multiple rib fractures, long bone fractures, shorter and flattened vertebral bodies, and narrow pelvis. Survivors show a moderately severe OI type IV outcome. In particular, mice with non-lethal phenotypes experience growth deficiency, lower bone mineral density (BMD), hypermineralization of the skeleton, bone weakness, brittleness and deformity, thereby reproducing human OI features (Kozloff et al., 2004).

The *Col1a2^+/G610C^*, often referred to as *Amish* mouse, is a knock-in murine model that recapitulates an OI type IV phenotype caused by a heterozygous *Col1a2* Gly610Cys variant. The mutation in this mouse model reproduces the molecular defect described in a large pedigree belonging to the Old Order Amish, characterized by wide phenotypic variability ranging from severe to non-symptomatic (Daley et al., 2010). Heterozygous pups show growth retardation, bones that are more brittle and prone to fractures than wild-type (WT) mice, and are characterized by decreased BMD, reduced cortical thickness and fewer and more-dispersed trabeculae (Masci et al., 2016). Raman spectroscopy shows an elevated mineral–to–collagen ratio and a reduced collagen content in *Amish* femora compared to those in WT littermates. *Amish* mice were crossed into five different inbred strains, providing a unique tool to evaluate possible effects of genetic background on skeletal phenotype severity (Daley et al., 2010). Indeed, this work made it clear that the mutation effects on whole-bone fracture susceptibility is influenced by individual strain-specific genomic factors that are reflected in bone size, shape and, possibly, the regulation of bone metabolism (Daley et al., 2010).

The *Col1a1^+/^*^Jr^*^t^* mouse, generated by N-ethyl-N-nitrosourea (ENU) mutagenicity, carries a heterozygous donor splice site mutation (+2) originally reported in an OI type I patient. The mutation results in skipping of the *Col1a1* exon 9, yielding an 18–amino acid deletion in the helix domain of the α1(I) chain (Chen et al., 2014). *Col1a1^+/^*^Jr^*^t^* mice display a more-severe phenotype than the human OI type I, with lower BMD, reduced bone volume (BV) and increased fractures compared to WT littermates (Chen et al., 2014).

Recently, zebrafish (*Danio rerio*) has emerged as an extremely interesting organism for modelling skeletal diseases owing to the conservation of bone-forming and -resorbing cells between humans and *D. rerio*, and also because of the pattern of zebrafish gene expression during bone cell differentiation and the type of ossification (Dietrich et al., 2021; Tonelli et al., 2020). To date, several zebrafish models for dominant OI are available (detailed below). Their main limitation is associated with the composition of collagen type I since, owing to the duplication of the *Col1a1* gene during teleost fish evolution, *D. rerio* carries three *Col1* paralogs (Gistelinck et al., 2016). The three collagen chains α1, α3 and α2 are encoded by *col1a1a*, *col1a1b* and *col1a2*, respectively, and present in adult skin, scales and bone in a ratio of 1:1:1 (Gistelinck et al., 2016).

Indeed, two zebrafish mutants that are heterozygous for a premature stop-codon mutation in either *col1a1a* (p.Gly1179Ter) or *col1a1b* (p.Cys68Ter) – with ‘Ter’ indicating the termination codon – do not reveal any skeletal abnormalities, probably because of the functional redundancy between the two chains. Like humans, zebrafish with a heterozygous *col1a2* (p.Ala154CysfsTer23) null mutation are asymptomatic (Gistelinck et al., 2018).

The best-characterized OI zebrafish model carrying a qualitative defect is *Chihuahua* (*Chi*/+), identified in a large ENU mutagenesis screening for skeletal defects (Fisher et al., 2003). *Chi*/+ is heterozygous for a glycine substitution (Gly574Asp based on the human legacy system; p.Gly736Asp based on the systematic *D. rerio* amino acid sequence) in the triple- helix region of the α1(I) chain, showing a severe and dominant OI phenotype that resembles OI type III, and results in decreased body length, bone fragility and delayed mineralization. Micro-CT 3D reconstructions of *Chi/+* zebrafish revealed fracture callus formation, deformation in the ribs and heterogeneous mineralization, particularly along the spine (Gioia et al., 2017). Significantly limited vertebral body length and thickness are found in their precaudal vertebrae compared to WT fish, together with significantly reduced BV, BMD and bone thickness (Fiedler et al., 2018).

More recently, a large ENU mutagenesis screening has yielded four additional OI zebrafish mutants carrying heterozygous glycine substitutions in type I collagen, namely *dmh13* (α1-Gly931Arg, p.Gly1093Arg), *dmh14* (α1-Gly981Glu, p.Gly1144Glu), *dmh15* (α2-Gly802Asp, p.Gly882Asp) and *dmh29* (α3-Gly958Asp, p.Gly1123Asp). These mutant strains have been characterized as valid models for OI with reduced length, vertebral deformity, hypermineralization and frequent fractures (Gistelinck et al., 2018).

Further characterization of these models will help to answer the numerous open questions regarding the genotype–phenotype relationship in OI.

## Extracellular or intracellular environment: which has a larger impact on phenotype?

Do chain type and/or position of collagen I sequence variants affect a defined severity pattern owing to extracellular or intracellular effects? To answer this puzzling and unsolved question is crucial in the attempt to improve the scientific understanding of OI and any therapeutic developments.

### Extracellular contributions to phenotypic variability

The hallmark clinical feature of OI, i.e. bone fragility, is caused by low bone mass and abnormalities in bone material properties. In healthy bone tissue, the hydroxyapatite crystals deposit in a well-organized manner on the organic ECM, >90% of which is constituted by collagen type I – the reason why its relevance in determining OI outcome was considered first. Before mineralization can be initiated, the collagen molecules self-assemble in fibrils in the extracellular space. These heterotypic collagen fibrils interact with cell-surface and ECM proteins, contributing to overall tissue structure and homeostasis. Glycine substitutions or exon skipping events can delay the tight folding of the triple helix, leading to excessive post-translational modifications (Cabral et al., 2006) and 3D structure distortion of collagen that are ultimately responsible for altered fibril assembly in the ECM (Prockop et al., 1993). However, the consequences of these modifications are complex. Increased hydroxylysine (Hyl) – i.e. a post-translational modification of collagen I – does not grossly affect the arrangement of intrafibrillar collagen molecules, as the number of crosslinks is similar in OI and control bone (Vetter et al., 1993). However, an increased number of mutant molecules with altered crosslinks has been reported on the surface of OI fibrils, suggesting a selective distribution of normal and structurally abnormal molecules, although distribution is independent of the clinical severity (Bank et al., 2000). Of note, increased levels of Hyl and glycosylated Hyl in OI result in wider-spaced individual collagen molecules within the fibrils, owing to the bulkier sugar rings that also affect fibrillogenesis as they reduce fibril diameter; but – again – no association with OI type has so far been reported (Bishop, 2016; Nijhuis et al., 2019).

As assessed by SDS-PAGE analysis, the three classic OI murine models, *Brtl*, *Amish* and *Col1a1^+/^*^Jr^*^t^*, reveal abnormalities in collagen modification. Lethal and non-lethal *Brtl* mice carry smaller fibrils and show a disordered collagen molecule assembly (Kuznetsova et al., 2004; Wallace et al., 2011). Similarly, reduced fibril diameter has also been described in *Col1a1^+/^*^Jr^*^t^* mice (Chen et al., 2014), whereas increased crosslink maturity in *Amish* mice has been linked to disorganization or over-modification of collagen molecules in bone (Masci et al., 2016).

Collagen fibril alignment is disturbed in the bones of OI patients, as biopsies show less-ordered and fewer lamellar fibrils than in controls, although without marked differences among OI types (Rauch et al., 2000). Similar to humans, altered fibril organization has been reported in *Brtl* and *Amish* mice, although *Col1a1^+/^*^Jr^*^t^* mice have normal fibril alignment (Blouin et al., 2019; Chen et al., 2014). To date, no information on fibril size or alignment is available for the zebrafish models of dominant OI.

It is accepted that the collagen type I triple helix domain includes both tighter and looser (less stable) regions, although their locations, properties and role in determining function are still poorly defined (Makareeva et al., 2008). Makareeva and colleagues hypothesized that more-flexible regions are more permissive compared with incorporation of bulkier amino acid without having major effects on folding and secretion or devastating extracellular consequences. However, in the same paper, the authors identified several lethal variants within a C-terminal flexible region (amino acids 676–832) (Makareeva et al., 2008). Interestingly, among the classic zebrafish models of dominant OI, *dmh15*, which carries a mutation in α2-Gly802Asp positioned within the C-terminal flexible region, shows a very severe outcome (Gistelinck et al., 2018). Furthermore, the splice site mutation in the *Col1a1^+/^*^Jr^*^t^* mouse is positioned within the highly stable N-anchor domain (Gly1-88), in which sequence variants were reported to be associated with an OI and/or Ehlers-Danlos syndrome (EDS) outcome. Consistent with this expectation, this mouse model exhibits a moderate OI type IV and/or EDS phenotype (Chen et al., 2014).

Thus, impaired collagen fibril structure that can alter its interaction with biologically important molecules, e.g. proteoglycans, integrins, extracellular glycoproteins, such as osteonectin and alkaline phosphatase, cytokines and cell adhesion molecules, might be a significant contributor to OI severity. In addition to impaired binding, simply changing the ratio of non-collagenic to collagen molecules can alter the ECM, consequentially having a detrimental effect on the tissue (Bishop, 2016). MBLR1, MLBR2 and MLBR3 have a high number of ligand-binding sites, with many ligands competing for identical sites (Di Lullo et al., 2002; Sweeney et al., 2008). Sequence variants in any of these regions may impair collagen ability to interact with other molecules, thus undermining ECM organization, cell adhesion and other essential processes. MLBR3, located at the C-terminal end of the 3D collagen fibril structure, represents the region highly exposed to the microenvironment and is, thus, freely accessible to cells and macromolecules. Indeed, variants in MLBR3 are mostly associated with lethal OI phenotypes (San Antonio et al., 2020).

Of note, *in vitro* cultures of bone cells from individuals with either lethal or non-lethal OI show a clear reduction in the levels of some ECM proteins, such as total proteoglycan, biglycan, decorin and osteonectin (Fedarko et al., 1995a). Unfortunately, the OI-causative sequence variants have not yet been determined for these individuals, preventing more precise genotype-phenotype investigation. Interestingly, the bone tissue of a molecularly uncharacterized perinatal-lethal OI type II individual, completely lacked osteonectin, a glycoprotein highly enriched in bone and involved in initiating mineralization and promoting formation of mineral crystals (Fedarko et al., 1995b). More recently, reduced decorin and increased fibrillin-1 expression were reported in primary fibroblasts from individuals with lethal OI (Bini et al., 2021). Bone proteome profiling of the *Brtl* murine model supports the hypothesis that additional alterations in ECM composition in the presence of the identical collagen mutation may contribute to the different outcomes. Indeed, *Brtl* mice with lethal outcome show reduced expression of the bone matrix proteins matrilin 4, microfibril-associated glycoprotein 2 and thrombospondin 3 compared to surviving animals (Forlino et al., 2007b). As *Brtl* is maintained on a mixed genetic background, it would be informative to investigate whether the OI-causing variants are directly responsible for different sets of proteomic changes or whether these alterations occur because of epigenetic changes in the mixed background that combine with the common collagen variant to produce different outcomes.

A common feature in dominant OI also not correlated to disease severity is bone hypermineralization (Roschger et al., 2008). Indeed, increased mineral plates with altered size and elevated density are present among fibrils that are less consistent in size and shape in OI bone compared with those in healthy bone. They are looser, allowing more water and minerals between and within them, causing hypermineralization and increased brittleness (Fratzl-Zelman et al., 2014; Roschger et al., 2008). Furthermore, the thinner mineral plates in OI bone are sometimes less well homogeneously aligned and have an altered phosphate-to-carbonate ratio (Bart et al., 2014; Fratzl et al., 1996; Mähr et al., 2021). The misalignment of mineral plates in OI bone is also reflected in the decreased bone density, as determined by dual-energy X-ray absorptiometry (DXA), of individuals with OI – whose bone is, in fact, hypermineralized. Increased mineral-to-matrix ratio has also been reported in all three classic OI murine models *Brtl*, *Amish* and *Col1a1^+/^*^Jr^*^t^* (Daley et al., 2010; Kozloff et al., 2004; Roschger et al., 2014).

The increased fragility of OI bones depends on several parameters. These include the entire amount of bone material and/or mass, and the size and shape of the entire bone. The microarchitecture of cortical and trabecular bone is also crucial, particularly the cortical width and porosity, trabecular BV per tissue volume, and the trabecular number, i.e. the number of trabeculae per unit of length, and thickness. Moreover, the intrinsic properties of the bone material and bone matrix mineralization, the constitution of the organic matrix, and the interactions between mineral and organic phases also determine bone fragility in OI (Bouxsein and Seeman, 2009; Wagermaier et al., 2015). It is impossible to assess all these measurements in a clinical setting but animal models allow researchers to perform biomechanical tests that take into consideration both structural and material properties in predicting fracture risk. In all classic OI murine models, mechanical testing revealed increased propensity to fracture in response to decreased force as well as bone brittleness, which is defined as decreased post-yield displacement (Daley et al., 2010; Kozloff et al., 2004; Roschger et al., 2014). Interestingly, crossing *Amish* mice with various inbred strains yielded different parameters of bone geometry biomechanics, supporting a role of the genetic background in modulating skeletal properties and, thus phenotypic severity (Daley et al., 2010).

In this respect, an appealing tool to dissect OI phenotypic variability is the recent characterization of several OI zebrafish models that carry glycine substitutions (Gistelinck et al., 2018). Although still limited in their coverage of pathogenic variants identified in patients, zebrafish offer the advantage of providing models of five different glycine substitutions at various positions within different α(I) chains. Interestingly, phenotypic variability between the five mutants, as well as in the presence of one identical mutation, has been described. Variants carried by *dmh13*, *dmh14* and *dmh29* zebrafish occur in a collagen type I region that is homologous with amino acids 910–955 of MLBR3 in human collagen, for which no patient sequence variants have been reported to date. *dmh15* zebrafish carry a glycine substitution in the MLBR2 of collagen type I. Similar to reports from patients with variants in this site, the phenotype associated with this substitution is extremely severe and characterized by heavily distorted, misshapen and hypermineralized axial and cranial skeletons (Gistelinck et al., 2018). Of note, collagen analysis showed different levels of collagen over-modification in the various zebrafish mutants, in which a gradient of post-translational collagen modification correlated with the position of the sequence variant within the collagen helix (Gistelinck et al., 2018).

### Intracellular contributions to phenotypic variability

In the last decade, the role of the intracellular environment in modulating the OI skeletal phenotype has gained increasing attention. However, our understanding of the role affected collagen chains have, and of the type and/or position of the variant is still limited and requires further investigation (Makareeva et al., 2011; Marini et al., 2017). Several studies have used patient-derived and animal model samples, showing that the uncoupling of bone formation and bone resorption is linked to impaired osteoblast activity (Marini et al., 2003; Rauch et al., 2000). The observed partial retention of misfolded type I collagen in the ER triggers a complex stress response that impairs cell homeostasis (Bateman et al., 1986; Chessler and Byers, 1993; Forlino et al., 2007a; Lamandé et al., 1995; Lisse et al., 2008; Mirigian et al., 2016; Zhang et al., 1997). Similar findings have been described in *Brtl*, *Amish* and *Col1a1^+/^*^Jr^*^t^* mice (Chen et al., 2014; Mirigian et al., 2016; Uveges et al., 2008). The activation of canonical and non-canonical unfolded protein response (UPR), macroautophagy and ER exit sites (ERES)-associated unconventional autophagy, and apoptosis have been demonstrated in fibroblasts and osteoblasts from OI patients and animal models (Besio et al., 2019b; Besio et al., 2018; Garibaldi et al., 2021; Omari et al., 2018).

Upregulation of endoplasmic reticulum chaperone BiP (HSPA5), a key chaperone involved in regulation of the UPR, has initially been described in primary fibroblasts obtained from OI patients carrying proα1(I) and proα2(I) C-propeptide sequence variants but is absent in cells with glycine substitutions in the triple-helical regions (Chessler and Byers, 1993; Lamandé et al., 1995). More recently, the specific mutated chain has been linked to activation of specific branches of the UPR, i.e. whereas α1(I) sequence variants display signaling predominantly via protein kinase RNA-like ER kinase (PERK), leading to inhibition of translation and to apoptosis, patients who carrying sequence variants in α2(I) preferentially exhibit UPR via IRE1α signaling, which promotes protein folding and exerts a cytoprotective function (Besio et al., 2018). A deep molecular and biochemical investigation of *Brtl* and *Amish* murine primary osteoblasts confirmed upregulation of the UPR signaling branches via PERK and IRE1α, as well as enhancement of macroautophagy and apoptosis (Garibaldi et al., 2021). In *Brtl* cells, upregulation of autophagy- and apoptosis-related genes is morerobust than in *Amish* cells as, in the latter, upregulation of UPR genes prevails. A non-canonical autophagic pathway originating at ERESs has also been described in *Amish* osteoblasts, demonstrating the existence of different intracellular routes used to eliminate the intracellularly retained mutant collagen, which further complicates the understanding of the OI mechanism (Omari et al., 2018).

A proteomic analysis of *Brtl* bone samples confirmed the enhanced expression of ER chaperones and of proteins involved in protein degradation in mice with non-lethal outcomes as well as the preferentially increased expression of molecules involved in protein disruption and apoptosis in animals with lethal outcomes (Forlino et al., 2007b). These findings support a role for intracellular homeostasis in modulating phenotypic severity (Forlino et al., 2007b). Unexpectedly, proteins involved in cellular organization and, importantly, in cytoskeletal assembly are also differentially expressed in *Brtl* mice with different outcomes (Bianchi et al., 2012). Cytoskeletal abnormality in osteoblasts of calvarial and long bones is evident in mice with lethal phenotypes and, interestingly, negatively affects collagen deposition, cell proliferation as well as integrin and TGF-β signalling (Bianchi et al., 2015; Gagliardi et al., 2017). Specific downregulation of the intermediate filament vimentin, upregulation of the microtubule-organizing protein stathmin and the cytoskeletal regulator cofilin-1 in skin and bone samples from mice with a lethal phenotype explain the impaired formation and organization of the cytoskeleton (Bianchi et al., 2015; Pollard and Cooper, 2009). In addition, cytoskeletal disorganization compromises cell–cell and cell–ECM interactions because of a reduced number of integrin-based focal adhesions. A similarly disorganized cytoskeleton was confirmed in cells from an individual with lethal OI (Bianchi et al., 2015).

Perturbation of intracellular homeostasis is mirrored by enlargement of ER cisternae – as detected in human cells – as well as in all three classic OI murine models and the *Chihuahua* OI zebrafish. The increased size of the ER indicates that prolonged stress hampers cellular morphology and, importantly, might hinder osteoblast differentiation and activity (Gioia et al., 2012; Mirigian et al., 2016). Recently, proteomic analysis performed by using cell lysates of fibroblasts derived from three lethal (type II) and three non-lethal (type III) OI patients carrying glycine substitutions in either *COL1A1* or *COL1A2*, revealed 17 differentially expressed proteins exerting key effects on ECM structure and organization, cell signaling, as well as cell and tissue development and differentiation. Among these, the non-collagenous ECM protein decorin (DCN) was downregulated and fibrillin-1 (FBN1) was upregulated in individuals with lethal OI, whereas the cytoskeletal proteins palladin (PALLD1) and nestin (NES) were upregulated (Bini et al., 2021). The altered expression of these proteins might modulate individual phenotypes.

**Fig. 4.**
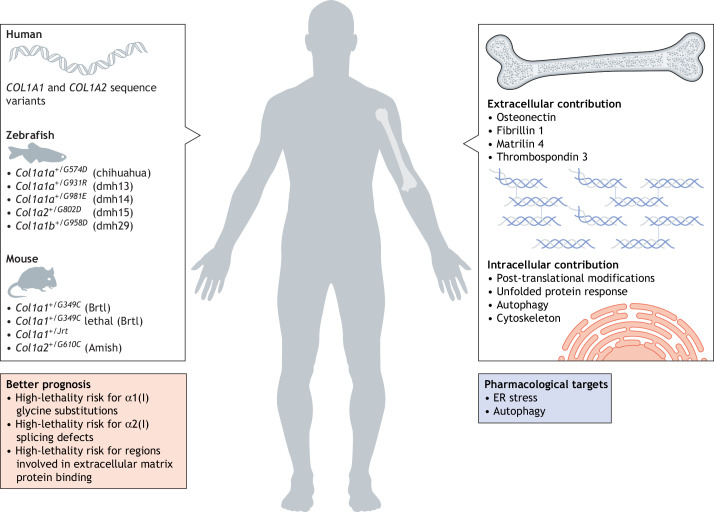
**Patient database records and animal models shed light on phenotypic variability of osteogenesis imperfecta (OI): insights on prognosis and new targets for therapy.** OI is a rare disease with variable severity. To understand the complex genotype–phenotype relationship in OI, researchers combine information from human mutation databases, and available zebrafish and murine OI models carrying dominant mutations in collagen I genes to dissect the contributions of extra- and intracellular factors to disease severity. These include non-collagen components of the extracellular matrix, as well as cellular and molecular processes, such as post-translational modifications (PTM), the unfolded protein response (UPR), and autophagy and cytoskeleton dynamics. These research efforts combined to understand phenotypic variability will improve OI prognosis and allow the identification of novel therapeutic targets.

Taken together, cellular homeostasis and extracellular abnormality both contribute to OI severity. The use of animal models has been, and will continue to be, crucial for the understanding of clinical variability; its deeper comprehension will shed light on further factors implicated in phenotype determination and will contribute to identifying new targets for therapy (Fig. 4).

## Conclusions

Here, we present an up-to-date analysis of the largest public OI database, and review the available literature on extracellular and intracellular effects of mutant collagen I that result from triple-helix α1(I) and α2(I) glycine substitutions or exon skipping variants.

Although the genetic cause of OI has long been established, we still lack a clear association between disease severity and the levels of mutation-induced collagen over-modification, collagen self-association, fibril alignment and organization, as well as bone hypermineralization. At least regarding glycine substitutions in the collagen I triple helix – considering their high frequency in both α(I) chains – additional approaches, i.e. beyond the molecular characterization of new patients, are probably needed to obtain further insight into OI phenotypic variability. To this end, animal models represent a useful approach. Interestingly, the biochemical analysis of cells and tissues from *Brtl* mice, the knock-in OI model in which the same glycine substitution yields both lethal and non-lethal outcomes, suggests that OI symptom and phenotype variation are related to abnormal interactions of mutant collagen helices with other ECM molecules, rather than to abnormal structure, physical properties or interactions among mutant helices (Forlino et al., 2007a; Kuznetsova et al., 2004). Furthermore, putative individual genetic variations of other ECM molecules might also modulate the OI outcome.

Interestingly, both *in vitro* and *in vivo* data from OI animal models indicate that intracellular homeostasis and cytoskeletal organization have a role in modulating OI severity (Forlino et al., 2007b). These data have been confirmed in OI murine and zebrafish models as well as in cells of OI patients (Besio et al., 2018; Bini et al., 2021; Garibaldi et al., 2021; Gioia et al., 2017). In particular, treatment with the chemical chaperone 4-phenylbutyrate (4-PBA), which reduces ER stress and UPR signaling, confirmed its role as modulator of the OI phenotype. 4-PBA decreases UPR and apoptosis *in vitro* – thereby restoring cell homeostasis, and also ameliorates mineralization and bone properties *in vivo* (Besio et al., 2018; Duran et al., 2022; Gioia et al., 2017; Scheiber et al., 2022). Other cell responses aimed at preserving cell activity, such as autophagy, will need to be investigated in animal models before they can be considered as therapeutic targets to treat OI.

The easy and relatively inexpensive generation and characterization of zebrafish OI models will provide a unique opportunity to screen for drugs that modulate these intracellular pathways. Furthermore, genetically tractable zebrafish models will allow the study of phenotypes both in the presence of different as well as identical sequence variants, and will provide a unique tool for the in-depth investigation of epigenetic and genetic modifiers of the disease.

OI is a rare disorder with a complex genotype–phenotype relationship. By integrating the careful clinical tracking of patients when using the *COL1A1* and *COL1A2* sequence variant databases with the work of *in vitro* and *in vivo* models, the field has recently begun to piece together this fascinating genotype–phenotype puzzle, thereby paving the way for accurate prognoses and the identification of effective treatments.
